# Impact and outcomes of the post-sophomore pathology fellowship at the University of Minnesota

**DOI:** 10.1016/j.acpath.2025.100211

**Published:** 2025-08-12

**Authors:** Katlin Wilson, Haley Fleckenstein, Cade Arries

**Affiliations:** Department of Laboratory Medicine and pathology, University of Minnesota Medical School, Minneapolis, MN, USA

**Keywords:** Medical student, Pathology, Pathology workforce, Post-sophomore fellowship, PSF, Residency

## Abstract

Post-sophomore fellowship in pathology programs have been utilized nationally as a tool to recruit medical students into the field of pathology; however, few articles document their outcomes particularly in regards to career outcomes, scholarly output, and qualitative feedback. The University of Minnesota Medical School offers a one-year post-sophomore fellowship in pathology for medical students with the aim of introducing medical students to the field of pathology and increasing knowledge of research methods; however, there is hesitancy among current medical students about delaying graduation by one year for fellowships. This study aims to evaluate the effectiveness of the post-sophomore fellowship in pathology at the University of Minnesota in terms of recruiting students into pathology specialties and fostering scholarly interest, as measured by career choices, publications, and interest in pathology. A retrospective analysis and survey revealed that approximately 55.6% of post-sophomore fellowship in pathology participants entered pathology specialties and that 69% of survey respondents indicated contributing to scholarly publications in the field of pathology during their post-sophomore fellowship in pathology year. Survey data revealed benefits of thepost-sophomore fellowship in pathology, including increased residency preparedness, letters of recommendation, and research engagement. Although the lack of a control group and potential selection bias limit the generalizability of these findings, our results suggest the post-sophomore fellowship in pathology model can attract medical students to pathology, foster research experiences, and better prepare students for residency.

## Introduction

Post-sophomore fellowship (PSF) in pathology programs have a long history at several institutions including the first program at the University of Rochester in the 1930s.^1^ By the 1950s, the University of California Los Angeles and University of Vermont established their PSF programs.^2^^,^^3^ In the 1970s, the American Board of pathology allowed the PSF year to count toward residency training, recognizing the program's value. However, as of 2001, the PSF no longer conferred credit for a year of residency as pathology training was reduced from five to four years.^4^

PSF programs aim to foster interest in and recruitment to pathology among medical students. Despite multiple studies evaluating and reporting on these programs, significant gaps persist in the literature. Early investigations, such as the Rochester student fellowship, indicated that fellows were substantially more likely to pursue academic or research-oriented careers than their peers.^1^ Similarly, another group reported that 36% of post-sophomore fellows at the University of Missouri, Columbia, pursued careers in pathology primarily in academic medicine.^5^ The University of California, Los Angeles (UCLA) report found that 19.3% of fellows in the UCLA PSF program became pathologists and that a substantial amount of these fellows went on to become UCLA's pathology residents. This was one of the earliest reports suggesting that a PSF program can impact pathology career selection.^2^ The University of Vermont's study reported both how many student fellows chose pathology and career milestones, where fellows were more likely to pursue careers in academic pathology and subspecialty training, providing new insights into types of pathology careers.^3^ These findings provide a valuable historical context for evaluating the long-term outcomes and ongoing relevance of PSF programs; however, they lack relevance to contemporary medical education, given significant curricular and accreditation changes over the years.

More recent studies have highlighted varied outcomes of the PSF programs.^6^^,^^7^ The University of Iowa published a recent study reviewing 21 years of data demonstrating that 43% of post-sophomore fellows entered pathology, accounting for 63% of medical school graduates entering pathology, and that one-third of former post-sophomore fellows occupied academic faculty positions.^7^ They concluded these outcomes show the PSF program as an effective tool in recruiting medical students to pathology. This study was one of the first to report student benefits and drawbacks from pursuing the PSF, filling a significant gap in the literature. Another group reported that among 20 national PSF programs, approximately 47.7% of participants eventually entered pathology residencies, suggesting effectiveness but indicating variability among institutions.^8^ The effectiveness of PSF programs in fostering scholarly activities among medical students was further demonstrated by another group, although their study emphasized quantitative outcomes, leaving qualitative aspects of student experiences unexplored.^7^ In addition to addressing the current challenges in recruiting pathologists, the importance of experiential exposure for medical students to pathology has been emphasized in the literature.^8^ A recent study also highlighted how hands-on experiences during clinical rotations significantly influence medical students’ interest in pathology as a career.^9^ This reinforces the value of programs like the PSF in providing direct exposure to pathology practice.

Despite these contributions, the current literature remains somewhat limited. Most studies do not address the comprehensive qualitative outcomes such as motivations for participation, personal or professional hardships, and subjective experiences related to program impact on career decision-making, including the more nuanced benefits/drawbacks outside of outcomes like residency placement. There remains more to be explored in further understanding barriers to student recruitment to the PSF program, especially within the context of increasing cost of medical education with decreased exposure to pathology in modern medical school curriculum.^10^

Our study directly addresses these existing literature gaps by providing quantitative career outcome and qualitative insights regarding participant experiences at the University of Minnesota PSF program. By combining objective metrics of success with subjective participant feedback, our study aims to offer a balanced assessment of the program's overall impact.

The pathology workforce may face a critical shortage as retirements increase and emerging healthcare demands intensify, which could cause a gap that cannot be met by the current number of trainees.^11^ PSF programs exist today, in part, to address this gap by fostering interest in and recruitment to pathology among medical students, with student costs including deferring graduation from medical school by one year. With modern medical curricula providing reduced exposure to pathology, PSF programs aim to address both the shortage of pathologists in the healthcare workforce and the diminished formal pathology instruction in medical schools by providing trainees hands-on experiences on pathology services.^12^

## Materials and methods

The study is designed to obtain objective data on the PSF program at the University of Minnesota's Department of Laboratory Medicine and pathology. Outcome measures include the benefits and limitations of the program, its ability to recruit medical students into pathology specialties, and to foster interest in academic careers. The study was reviewed and approved by the University of Minnesota Institutional Review Board (STUDY00020852).

### Practice setting

The University of Minnesota's Department of Laboratory Medicine and pathology offers a PSF program aimed at medical students who have completed their preclinical years and are enrolled at any U.S. medical school.^13^ At the time of this publication, the University of Minnesota is one of 24 PSF programs offered out of almost 200 Doctor of Allopathic Medicine– and Doctor of Osteopathic Medicine–accredited medical schools.^14^, ^15^, ^16^, ^17^ The program has employed 31 post-sophomore fellows (averaging 1–2 per year) from 2005 to date. While the program has considered and enrolled both internal and external applicants, internal candidates are more commonly recruited. To address hesitancy around the extra year of medical school, the department works closely with students and medical school administration to facilitate an extended graduation timeline, allowing students to take advantage of learning experiences outside of the traditional curriculum without incurring additional tuition fees.^18^

Spanning a year, the PSF program emulates the roles and responsibilities typical of first-year pathology residents while still maintaining the level of supervision required for medical students in clinical rotations. The PSF program starts with a one-month “boot camp,” completed alongside the incoming first-year residents, followed by rotations in both anatomic and clinical pathology. The boot camp offers a variety of lectures and active didactic sessions to new learners covering the basics of histology, grossing, pathobiology, laboratory workflow, special studies, use of electronic medical record, as well as other onboarding materials. The post-sophomore fellows and new pathology residents also cover the autopsy service during the boot camp month, with a senior resident and attending, to practice grossing, histological interpretation, and clinical correlation. At the beginning of the PSF year, post-sophomore fellows are provided with a rotation syllabus, intended for pathology residents, containing the learning objectives and information on rotation specifics (An example schedule with rotation options is provided in Supplemental Material 1). Post-sophomore fellows are expected to review and satisfy the objectives and requirements typically assigned to residents while under a senior resident and attending supervision.

Post-sophomore fellows are integrated into the resident rotation schedule, though on-service sign-outs and procedural supervision remain at the discretion of attending pathologists and certified Pathologists’ Assistants. Competency is assessed on a rotation-by-rotation basis, using the same resident evaluation forms, and post-sophomore fellows remain under direct supervision for all responsibilities including grossing surgical specimens, autopsy services, and intraoperative consultations/frozen sections.

The program also incorporates time for post-sophomore fellows to engage in faculty-led translational research opportunities, many of which are presented at national annual pathology conferences such as the United States and Canadian Academy of pathology (USCAP), American Society for Clinical pathology, and Academy of Clinical Laboratory Physicians and Scientists (ACLPS). Research is initiated by the post-sophomore fellow, either by joining an existing project or creating their own, and can be completed throughout the year or during a research block. The goal is for the post-sophomore fellow to present or publish while in the fellowship, however, is not a requirement in the program. Similar to other PSF programs, participants receive a stipend and financial support to present at national conferences. Faculty are encouraged to submit tailored written feedback at the conclusion of each training block, aimed at steering the trainee's academic and professional development. Post-sophomore fellows are recognized with a certificate at the graduation ceremony within the Department of Laboratory Medicine and pathology.

### Post-sophomore fellowship in pathology outcomes

This study includes alumni who completed the PSF program at the University of Minnesota from 2005 to 2023. Residency and fellowship information, as well as publication data, was obtained from publicly available sources, including Google (Mountain View, CA), LinkedIn (Sunnyvale, CA), and Doximity (San Francisco, CA). Individuals who were still enrolled in medical school or for whom relevant data could not be identified through online searches were excluded. Key metrics from the data collected included post-PSF career path (residency/fellowship specialty and location), the number of research projects initiated during the PSF year, the number of publications within five years of completing the PSF, and the number of conference presentations on PSF-related work. Descriptive statistics were used to evaluate these quantitative data, and the Wilcoxon signed-rank test was used for matched-pair analyses.

### Post-sophomore fellowship in pathology experience survey

PSF in pathology alumni were surveyed through email about their experience, including potential benefits/hardships, interest in pathology as a specialty, relevance of skills learned in the program to chosen residency, publications/scholarly contributions during the PSF program, and likelihood of recommending the PSF program to others. Respondents had the option to submit the survey anonymously and provide additional written feedback. This qualitative feedback was analyzed for themes by grouping text into certain categories based upon topic. The survey was administered to all alumni in December 2023 and January 2024, after all participants had completed their PSF. The full survey tool is available as Supplemental Material 2.

## Results

### Post-sophomore fellow outcomes

Thirty-one medical students were enrolled in the PSF program from 2005 to 2023. Two were excluded as they were still enrolled in medical school at the time of this study, and two were excluded because data was not available. Twenty-seven alumni were included in the study after exclusion criteria.

Of the 27 program alumni included as participants in this study, 55.6% (n = 15) chose pathology residencies, and many of those residents pursued pathology subspecialty fellowships. A smaller proportion of participants pursued other specialties (n = 11; 40.7%), and one chose an alternate healthcare career (3.7%). The other specialties included general surgery, internal medicine, pediatrics, anesthesiology, diagnostic radiology, neurosurgery, and radiation oncology. Further data on specialty and program locations are shown in Table 1.

**Table 1:** Study participants’ residency specialties pursued by post-sophomore fellows and locations.

Residency specialty	Number of post-sophomore fellows
Pathology	15
Anesthesiology	1
Diagnostic Radiology	1
General Surgery	3
Internal Medicine	3
Neurosurgery and Radiation Oncology	1
Pediatrics	2
Physical Medicine and Rehabilitation (PMR)	1

Residency Institution	Number of post-sophomore fellows

**University of Minnesota**—Minneapolis, Minnesota	4
**Case Western Reserve University/University Hospitals Cleveland Medical Center**—Cleveland, Ohio	1
**HCA Healthcare LewisGale Hospital Montgomery**—Blacksburg, Virginia	1
**Hennepin County Medical Center**—Minneapolis, Minnesota	1
**Jackson Memorial Hospital**—Miami, Florida	1
**Mass General Brigham**—Boston, Massachusetts	1
**Medical College of Wisconsin**—Milwaukee, Wisconsin	1
**MetroHealth Medical Center**—Cleveland, Ohio	1
**Mayo Clinic**—Rochester, Minnesota	2
**Mount Sinai**—New York, New York	1
**Northwestern McGaw Medical Center**—Chicago, Illinois	1
**St. John Providence Health System**—Detroit, Michigan	1
**State University of New York Downstate Health Sciences University**—Brooklyn, New York	1
**Truman Medical Center**—Kansas City, Missouri	1
**University of California, San Francisco**—San Francisco, California	1
**University of Illinois, Chicago**—Chicago, Illinois	1
**University of Iowa**—Iowa City, Iowa	1
**University of Toledo**—Toledo, Ohio	1
**University of Medicine and Dentistry of New Jersey**—Newark, New Jersey	1
**University of Washington**—Seattle, Washington	1
**Vanderbilt University**— – Nashville, Tennessee	1

Outlined here are the different residency specialties chosen by former PSFs and also the different institution residency programs of the prior PSFs.

PSF: Post-sophomore fellowship in pathology.

All program alumni who completed pathology residency (n = 12, 3 currently in residency) pursued at least one pathology subspecialty fellowship, with an average of 1.5 fellowships per program alumni. Most common fellowships included surgical pathology (n = 4, 33%), cytopathology (n = 3, 25%), and hematopathology (n = 4, 33%).

### Former post-sophomore fellow experience survey results

For the survey, we identified emails for 24 of the 27 included participants. When analyzing survey data results, a participant count of 24 rather than 27 is used. The survey yielded a total of 13 responses (54% response rate). Respondents were asked to rank their interest in pathology as a specialty on a scale from 1 to 5 before the post-sophomore fellowship and after the post-sophomore fellowship, with 1 being low and 5 being high. The average interest in pathology before and after the PSF program was 3.6 and 4.2, respectively, with an average delta of 0.6 (n = 13, p = 0.221). The results did not achieve statistical significance.

**Fig. 1:**
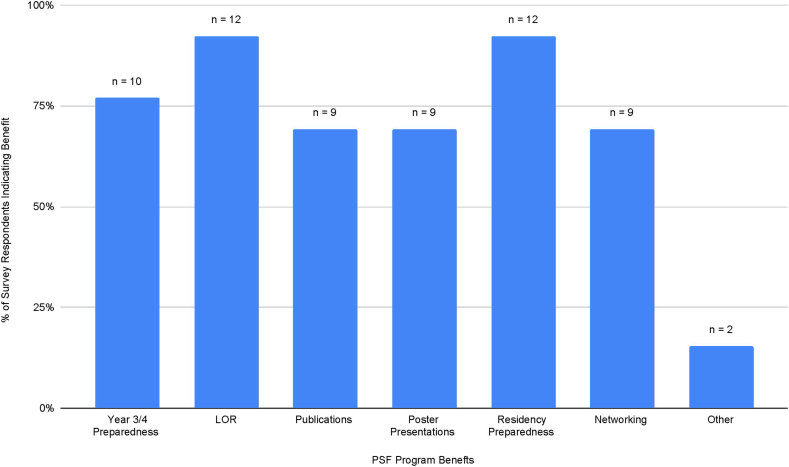
**Reported benefits of the PSF program.** Percent of response indicating each benefit. Respondents could select multiple answers. PSF: Post-sophomore fellowship in pathology.

Survey results indicated that benefits of the PSF program include acquiring meaningful letters of recommendation and residency preparedness, among others displayed in Fig. 1. Ninety-two percent of respondents found the PSF program helpful in applying for residency, regardless of specialty choice, and 100% would recommend the program to current medical students. Of the respondents who pursued pathology residences, 100% found the PSF program experience to be representative of their pathology residency experience.

To evaluate research outcomes and the programs' ability to foster interest in academic pathology, respondents were asked to provide information regarding research projects started during their PSF year (published during or within 5 years of finishing the PSF program). The average number of self-reported publications and poster presentations from research during their PSF year was 1.3 (without posters = 1.2). An average of 0.92 presentations were self-reported by former post-sophomore fellows including both national and interdepartmental conferences. Following a search of the various medical publication databases, we found that 66.67% of participants published in peer-reviewed journals up to five years post the PSF year, with an average of 3.1 publications per participant. Note, these are all publications, not exclusively the ones from their PSF year and may include publications early in residency. Additionally, 15.38% of the participants who responded to the survey have presented their research findings at national conferences such as USCAP and ACLPS, further substantiating the program's effectiveness in fostering a research-oriented mindset. Five out of 13 respondents are currently occupying or have occupied academic positions.

**Fig. 2:**
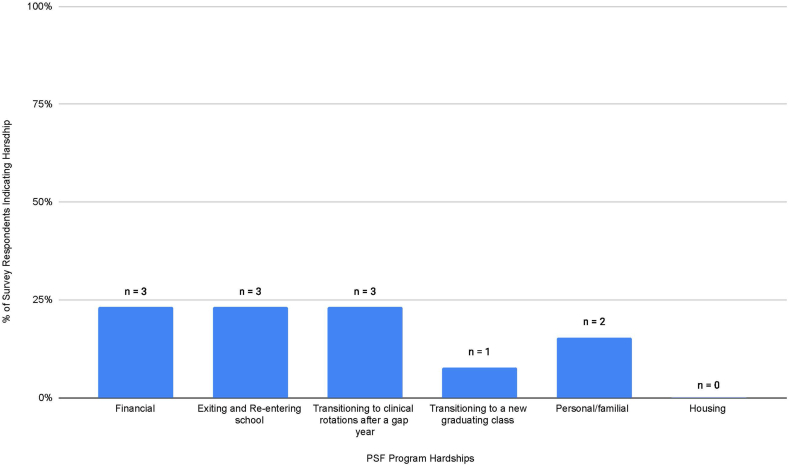
**Reported hardships of the PSF program.** Percent of response indicating each hardship. Respondents could select multiple answers. PSF: Post-sophomore fellowship in pathology.

The survey also collected information on potential hardships participants may have experienced to provide insights into current medical students. Fewer hardships were experienced than benefits (Fig. 2). The most common hardships listed include finances and the process of entering and exiting medical school.

An analysis of qualitative comments identified the following themes: career development, specialty clarity, confidence building, personal fulfillment, and research opportunities. The feedback centers on curriculum enhancement and need for consistent mentorship to further improve the program's impact. Full comments are listed in Supplemental Material 3.

## Discussion

Our findings contribute uniquely to the literature by explicitly incorporating qualitative participant feedback with quantitative career outcomes. While prior studies have shown variability in PSF program effectiveness in recruiting students to pathology, qualitative insights into participant experiences have generally been overlooked. This study shows how mentorship, financial considerations, and curriculum integration influence participants' perceived value and decisions regarding residency specialty choices.

Our results demonstrate that the PSF program at our institution may contribute to attracting medical students into the pathology specialty. More than half of the post-sophomore fellows went on to complete pathology residency, and those who completed pathology residency also pursued at least one subspecialty fellowship on average. Our results on residency location show a possible preference for staying near Minnesota when choosing residency or fellowship programs.

Our quantitative results were overall consistent with prior publications, where our institution's recruitment rate of 55.6% into pathology was similar to the average of 47.7% among 20 PSF programs reported by Naritoku et al.^19^ This highlights the continued utility of PSF programs in recruiting medical students to pathology, despite changes in requirements including the removal of the PSF program counting toward the first year of residency.^4^

The research engagement and the proportion of trainees presenting their work, either through national conferences or publications, illustrate another facet of the program's effectiveness. The emphasis on research within the PSF program enriched the post-sophomore fellows’ academic experience and contributed to the broader scientific community. However, many former post-sophomore fellows opted to not work in academia, which is lower than the estimated average of 59.8% reported by Naritoku et al.^19^ This may be unique to the program as collaborations with community partners are common.

The survey results provide key quantitative and qualitative data demonstrating benefits for both participants and the field of pathology. More than half of survey respondents (69%) indicated contributing to scholarly publications during their PSF year and overall showed an average increase in interest in pathology as a specialty. However, the average increase in interest in pathology of 3.6 (before fellowship) to 4.2 (after fellowship) on a 5-point scale could have been influenced by factors outside of the PSF program that have not been addressed. The interest in pathology results did not achieve statistical significance, which could be due to a small sample size. There also is a possibility of pre-selection bias, as those students entering the PSF program might be students already interested in pathology, or a recall bias. Benefits reported include obtaining letters of recommendation for residency application, increased residency preparedness, and enhanced understanding of pathology. Main challenges include financial issues, exiting and re-entering medical school, and transition to clinical rotations following the PSF year. Our program has adapted such that students are able to receive increased financial support, earn elective credits, and extend their medical training without incurring additional costs for their medical degree. The thematic analysis of participant commentary suggests that participants value both the personal and professional development facilitated by the PSF program and the benefit in applying and preparing for residency, along with the confidence it provides as individuals transition into their medical careers.

The program not only sets many students on the path to specialized pathology careers but also equips participants with essential skills and knowledge that they carry into their future medical endeavors including building their professional network and earning letters of recommendation from mentors. The career outcomes underscore the program's success as a recruitment strategy into pathology with 55.6% of alumni entering pathology residencies. The program also offers robust research opportunities as evidenced by 66.7% of participants publishing peer-reviewed articles within five years post the PSF program (average of 3.1 publications each). For those who have chosen other specialties, the PSF experience offered insights into disease mechanisms and diagnostic methodologies, as well as experience in patient care collaboration with the Department of Laboratory Medicine and pathology. Properly collaborating and consulting with pathology departments is a skill not typically taught in traditional medical curricula and can significantly improve patient care. For these reasons, participation in a PSF program may grant individuals a competitive edge for residency applications, with meaningful physician letters of recommendation, and may perform at a higher level in residency with a more complete understanding of pathology as an integral part of the clinical team.^10^

Nonetheless, our results must be interpreted cautiously due to methodological limitations, including potential pre-selection bias among students who might already possess an interest in pathology, recall bias in retrospective studies, and the limited generalizability due to small sample size. Future studies including multi-institutional prospective evaluations could further strengthen understanding of the broader impacts and effectiveness of PSF programs nationally.

Informal feedback from students who are hesitant or choose not to participate in the program share the unfavorable nature of delaying graduation by another year. Medical students who have completed the program shared the value of the fellowship in residency applications and interviews, as well as informed career discernment. pathology residency programs likely see the benefits of having a former post-sophomore fellow join their program based upon the sheer amount of knowledge gained during this experience; however, no data are published to know if post-sophomore fellows are favored in residency program rank lists. The current PSF shared a similar sentiment, in addition to valuing the change to focus on personal and professional development outside of the rapid pace and quick decision-making that is required by traditional clinical clerkship medical curricula. This change of focus was also a commonly echoed reason for choosing to do a PSF by former post-sophomore fellows when asked at a College of American Pathologists–sponsored webinar in 2023 hosted by former post-sophomore fellows discussing their experience for current medical students.^20^

We acknowledge that this study is limited by its retrospective nature and potential selection and recall biases. Additionally, publicly available data from program websites, online searches (Google, LinkedIn, and Doximity), and employee records may not always be up to date or accurate as such sources are not consistently maintained. This limitation could affect the completeness or accuracy of the information regarding alumni outcomes. We neither have accounted for long-term career trajectory changes nor have we compared PSF program alumni with a control group of medical students who did not undergo the fellowship. Additionally, we did not include data from the inception of the program as these data are not available and were prior to the formalization of the program in 2005. We did not collect information on how the participants heard about or were recruited into the PSF program; therefore, it is unclear whether past post-sophomore fellows were already interested in pathology. Our survey tool was not validated prior to use, a common limitation in survey-based research, which should be considered when interpreting the results. Despite these limitations, the approach of the study offers valuable insights into the effectiveness of the PSF program in encouraging careers in pathology and enhancing foundational knowledge.

To build upon and encourage interest in pathology as a specialty and appeal to a greater pool of applicants, we recommend offering additional provisions beyond the current stipend and educational expenses and minimizing the administrative burden in exiting and re-entering medical school. At the University of Minnesota students may opt to remain enrolled in the University during their FlexMD year to remain eligible for school health insurance and claim elective credits. The elective credits allow greater flexibility for time off for dedicated boards studying and away rotations/interviews during their clinical years. Recently, our undergraduate medical school curriculum has been updated with a shorter foundational/preclinical phase which will also provide new challenges to the PSF program, and we will continue to evaluate the program in the new curriculum. Moving forward, an exhaustive end-of-fellowship survey and exit interview will be conducted to gain more information related to our specific PSF in pathology.

## Conclusion

Our analysis supports the effectiveness of our institution's PSF in pathology program in achieving its intended goals and providing post-sophomore fellows with opportunities for personal and professional development as they advance in their medical careers. The program has been successful in attracting medical students to pathology and enhances the overall educational experience for participants. Future studies could expand on these findings by investigating the long-term career trajectories of alumni and comparing them to non–PSF program participants. Furthermore, gauging residency attitudes toward applicants who have completed the program would provide invaluable insights into those considering a PSF program. Based on the existing data, the PSF program shows how specialized training can serve as both an educational opportunity and a way to recruit medical students into pathology.

## Funding

This research received no specific grant from any funding agency in the public, commercial, or not-for-profit sectors.

## Declaration of competing interest

The author(s) declared no potential conflicts of interest.
