# Supplementary material for: Impact and outcomes of the post-sophomore pathology fellowship at the University of Minnesota
Source: Acad Pathol. 2025 Aug 12;12(3):100211. doi: 10.1016/j.acpath.2025.100211 (PMC12359211; doi:10.1016/j.acpath.2025.100211)
Supplement: Multimedia component 1 [file mmc1.docx]

**Supplemental Material 1:** One example of a post-sophomore fellow schedule, and all available elective rotations that can be chosen by the PSF to create their own custom experience.

| PSF | July | August | September | October | November | December | January | February | March | April | May | June |
| --- | --- | --- | --- | --- | --- | --- | --- | --- | --- | --- | --- | --- |
| 1 | Intro #AP | Transfusion #CP | Autopsy #AP | Surg Path GI #AP | Cell Therapy #CP | Surg Path Dermpath #AP | Research | Cytology #AP | Molecular #CP  Surg Path ENT/Thoracic #AP | Heme #CP | N/A | N/A |

List of Electives:

- Autopsy
- Bone and Soft Tissue Pathology
- Breast Pathology
- Cytopathology
- Dermatopathology
- ENT-Thoracic Pathology
- Genitourinary Pathology
- GI Pathology
- Gynepathology
- Neuropathology
- Pediatric Pathology
- Renal Pathology
- Research
- Transplant Pathology
- Blood Bank and Transfusion Medicine
- Clinical Chemistry
- Coagulation
- Cytogenetic
- Hematopathology
- Immunology / HLA
- Microbiology
- Molecular Genetic Pathology
- Cellular Therapy
- Digital Pathology
- Informatics
