# Supplementary material for: Impact and outcomes of the post-sophomore pathology fellowship at the University of Minnesota
Source: Acad Pathol. 2025 Aug 12;12(3):100211. doi: 10.1016/j.acpath.2025.100211 (PMC12359211; doi:10.1016/j.acpath.2025.100211)
Supplement: Multimedia component 2 [file mmc2.docx]

**Supplemental Material 2:** Post-Sophomore Fellowship in Pathology Experience Survey Text.

Post-Sophomore Fellowship in Pathology Experience Survey

We are conducting research on the experience of Post-Sophomore Fellows from the University of Minnesota to evaluate the program and improve the experience for PSFs to come. Please take some time to fill out this survey regarding your experience as a PSF. We appreciate your time and response!

For questions, comments, or concerns, please reach out to Dr. Cade Arries at arrie003@umn.edu.

1. Name: *
2. If you are open to being contacted in the future regarding your PSF experience, please provide your email below (optional):
3. Residency Specialty
4. Completed Fellowship Specialty(s) (if any)
5. Please select all of the below options which you experienced as benefits of the PSF program:
   1. Years 3-4 Preparedness
   2. Letters of Recommendation
   3. Publications
   4. Poster Presentations
   5. Residency Preparedness
   6. Networking
   7. Other…
6. Please indicate the number of publications that were started during your PSF year (if none, type 0). If known, please include the DOI.
7. Please indicate (if any) the conference(s) you were able to present at during your PSF year or as a result of your work as a PSF. If you were not able to, please enter n/a.
8. Did you experience hardships during your PSF year? (check all that apply)
   1. Financial
   2. Exiting and entering school
   3. Housing
   4. Transitioning to clinical rotations after a gap year
   5. Personal/familial
   6. Other…
9. Was your PSF year helpful when completing residency applications?
   1. Yes
   2. No
   3. Maybe
   4. Other…
10. Was your PSF experience representative of pathology residency?
    1. Yes
    2. No
    3. Maybe
    4. I did not complete a pathology residency
    5. Other…
11. If you did not complete a pathology residency, were the skills and information you learned during your PSF year applicable to your chosen residency?
    1. Yes
    2. No
    3. Maybe
    4. Other…
12. Would you recommend the PSF experience to current students?
    1. Yes
    2. No
    3. Other…
13. Are you currently working or have you worked in an academic job position post-PSF year?
    1. Yes
    2. No
14. Please rate your interest in pathology as a specialty before your PSF year:

Low <1> <2> <3> <4> <5> High

1. Please rate your interest in pathology as a specialty after your PSF year:

Low <1> <2> <3> <4> <5> High

1. Is there anything else you would like to share about your PSF experience? Please feel free to use this space to elaborate on any questions above.
