# Supplementary material for: Impact and outcomes of the post-sophomore pathology fellowship at the University of Minnesota
Source: Acad Pathol. 2025 Aug 12;12(3):100211. doi: 10.1016/j.acpath.2025.100211 (PMC12359211; doi:10.1016/j.acpath.2025.100211)
Supplement: Multimedia component 3 [file mmc3.docx]

**Supplemental Material 3:** Qualitative feedback responses from survey to the indicated question.

| Is there anything else you would like to share about your PSF experience? Please feel free to use this space to elaborate on any questions above. | |
| --- | --- |
| Participant #1 | The PSF program is excellent |
| Participant #2 | My PSF year was sooooo helpful for residency. I feel like I entered residency with less anxiety and more base knowledge than my fellow residents. The connections I made and knowledge I gained that year were second to none. It also helped with my interviews and significantly helped me get into my top program. Ten out of ten, would recommend. |
| Participant #3 | My PSF year was very valuable - it helped me figure out that pathology was a possible career path, helped me understand my interests better, and helped me realize I would be happier with a different future career path. It helped with my confidence. I still pull experiences from my PSF year to directly help with patient care. I am a much better physician as a result of completing the PSF year. It has created occasional questions about "why was your medical school time extended" that are easily answered. People outside of pathology and academia are less likely to be familiar with the PSF model. |
| Participant #4 | Please have PSF student do their AP rotations before their CP rotations.  Please have the PSF student do a 10 minute presentation every few weeks AND GIVE THEM FEEDBACK. Guide them with the presentation if no previous experience in pathology  Please have them learn cutting slides on the cryostat  Please have them rotate through histology so they know what happens behind the scenes before they get their slides.  Having quarterly detailed check-ins with them to gage progress will be a good idea.  Have them study for unknown test in cytology and take the test |
| Participant #6 | My PSF year not only helped me succeed during my clinical rotations and pathology residency, but also increased my confidence that I was choosing the right specialty for me. It also helped me better understand what I was looking for in a residency program. I would highly recommend the PSF program. |
| Participant #7 | It was an absolutely great advantage to have for me, especially continuing on to pathology for residency. The benefits described in the survey were all present as I moved on to residency.  I want to defend against some of the potential hardships that were described. 1. Financial burden: this was not felt for me, there was a stipend included in the experience and all my student loans were able to be put on pause thanks to the Flexible MD program at the University of Minnesota. So relative to the negative cash flow of medical school there was a modest positive cash flow during this time. 2. Returning to clinical training: I was worried at first that I would lose the connections with my fellow classmates. However, the reality was that I still was on clinical rotations with my classmates, when I was a 3rd year they were 4th years. So we still crossed paths. 3. Personal/Family: It was a great experience overall, and allowed me to have a personal life. Including that fact that I got married during this experience as well.  Research: While I specifically got a single case report published as a PSF, I did get embedded in a much larger project with prostate cancer. A big radiology and pathology collaboration that spanned through the rest of medschool and through pathology residency. It did result in publications, but these publications were later in residency and after. So I did not include them in the count here. I did have oral and poster presentations on those topics as well.  Overall, the exposure and pathology and involvement really helped solidify my desire to be a pathologist. Specifically for me, one of my last rotations was Transfusion Medicine. And I am always reflecting on this when I am talking to pathology applicants, I think that being a confident PSF who was almost a year in, really enhanced my experience in Blood Banking. And as a result, that is where my career path took me. |
| Participant #9 | Truly invaluable, I learned a lot during that year |
